# Clinical Experience With an L-Proline–Stabilized 10 % Intravenous Immunoglobulin (Privigen®): Real-Life Effectiveness and Tolerability

**DOI:** 10.1007/s10875-014-0070-z

**Published:** 2014-07-01

**Authors:** Morna J. Dorsey, Viet Ho, Mohsen Mabudian, Pere Soler-Palacín, Nerea Domínguez-Pinilla, Radha Rishi, Rahul Rishi, Duane Wong, Mikhail Rojavin, Alphonse Hubsch, Melvin Berger

**Affiliations:** 1 Department of Pediatrics, University of California, San Francisco, CA USA; 2 Moffitt Cancer Center, Tampa, FL USA; 3 Department of Allergy and Clinical Immunology, Beaver Medical Group, Inc., Redlands, CA USA; 4 Pediatric Infectious Diseases and Immunodeficiencies Unit, Hospital Universitari Vall d’Hebron, Barcelona, Spain; 5 Department of Pediatrics, Hospital 12 de Octubre, Madrid, Spain; 6 Arizona Allergy Associates, Phoenix, AZ USA; 7 CSL Behring LLC, King of Prussia, PA USA; 8 CSL Behring, Berne, Switzerland; 9 Pediatric Allergy-Immunology/BMT, University of California San Francisco, 1466 4th Avenue, Medical Research Building 4 (MRIV), Room 114B, Campus Box 0107, San Francisco, CA 94143-0107 USA

**Keywords:** Primary immunodeficiency, PID, Secondary immunodeficiency, IVIG, Privigen®, IgPro10, Clinical practice

## Abstract

**Purpose:**

This retrospective study evaluated the effectiveness and tolerability in clinical practice of an L-proline–stabilized 10 % intravenous immunoglobulin (IVIG; Privigen®) in patients with primary (PID) or secondary immunodeficiency (SID).

**Methods:**

Patients from 6 centers in Europe and the US were treated with individually determined regimens of Privigen® for ≥3 months. Serum immunoglobulin G (IgG) trough levels, annualized rates of infection, hospitalization and antibiotics use, and the incidence of adverse events (AEs) were analyzed.

**Results:**

Of 72 patients, three infants with severe combined immunodeficiency (SCID) were analyzed separately. The remaining 69 patients (52.2 % male; median age 38 years [range: 0.1–90.0]) with PID (82.6 %) or SID (17.4 %) received a mean (±standard deviation) Privigen® dose of 532 ± 250 mg/kg/month resulting in trough serum IgG levels of 407–1,581 mg/dL (median: 954 mg/dL). Ten patients (14.5 %) experienced 11 serious bacterial infections over 22.0 ± 15.0 months of treatment (0.087 events/patient/year, upper one-sided 99 % confidence interval: 0.170), the most common being pneumonia (11.6 %). The rates for any infection and hospitalization were 1.082 events/patient/year and 3.63 days/patient/year, respectively. Two patients with severe disease accounted for 303 of 460 hospital days. Across all 72 patients, 13 (18.1 %) patients experienced AEs, including 10 (13.9 %) patients with AEs at least possibly related to Privigen®, including headache (8.3 %), fever, and chills (2.8 % each). No related serious AEs were reported. One infant with SCID died due to severe viral infection.

**Conclusions:**

Despite the heterogeneous population, effectiveness and tolerability of Privigen® in clinical practice closely matched those reported in clinical studies.

## Introduction

Immunoglobulin G (IgG) replacement therapy, administered either intravenously (IVIG) or subcutaneously (SCIG), is the standard of care for patients with primary (PID) and secondary immunodeficiencies (SID). IVIG, and in some cases SCIG, are also used for the treatment of autoimmune conditions, such as immune thrombocytopenia, chronic inflammatory demyelinating polyneuropathy, Guillain–Barré syndrome and Kawasaki disease [1–5].

Privigen® (IgPro10; CSL Behring, Berne, Switzerland) is a 10 % liquid preparation of polyclonal human IgG for intravenous administration, stabilized with L-proline [6]. Privigen® is indicated as replacement therapy for PID including, but not limited to, congenital agammaglobulinemia, common variable immunodeficiency (CVID), X-linked agammaglobulinemia (XLA), Wiskott-Aldrich syndrome (WAS), and severe combined immunodeficiencies (SCID) [7]. In the EU, Privigen® is also indicated for the IgG replacement therapy in some patients with SID. Clinical studies have demonstrated the efficacy and tolerability of Privigen® in both adults and children with PID [8–10]. In clinical studies, administration of Privigen® every 3 or 4 weeks helped decrease the risk of recurrent infections and resulted in low annualized rates of serious bacterial infections (SBIs), any infections, hospitalization and days missed from school/work [8, 9]. Privigen® is well tolerated at infusion rates up to 8 mg/kg/min (4.8 mL/kg/h), with many patients tolerating infusion rates up to 12 mg/kg/min (7.2 mL/kg/h) [8–10]. Privigen® has been used in clinical practice since its registration in 2009 leading to accumulation of sufficient real-life effectiveness and safety data.

Registration studies of IVIG products are usually conducted in relatively homogenous, carefully selected patient populations with well-defined forms of PID receiving standardized treatment [8–12]. Here we report the real-life effectiveness and tolerability of Privigen® in clinical practice in 6 clinics located in Europe and in the United States (US). This retrospective cohort analysis (chart review) included patients with a wide variety of PID and SID diagnoses, treated according to local practice. Detailed data were obtained for most of the patients, with exceptions as noted in the text.

## Methods

### Patients and Data Collection

Patients of all ages who have received Privigen® for the treatment of PID or SID for at least 3 months in clinical practices in Europe and the US were eligible for this study. The Privigen® treatment schedule and doses were determined individually at the discretion of the investigators and according to the patients’ conditions and local practices. Information about dosage and regimens of Privigen® and other IgG products was collected retrospectively using a predefined data collection template. This study was conducted in accordance with the International Conference on Harmonisation Good Clinical Practice guidelines, and the Declaration of Helsinki (2008 version). The protocol of the study and all other study documents were approved by the relevant independent Ethics Committees or Institutional Review Boards.

### Effectiveness and Tolerability Assessments

Infant patients with severe combined immunodeficiency (SCID) were excluded from the main analysis and analyzed separately, due to potential lack of cell-mediated immunity leading to extremely severe phenotypes (see also Results and Discussion). In the remaining patients, demographics and the effectiveness of Privigen® were analyzed for patient subgroups based on age at the start of Privigen® (age classes): infants (0–2 years old [y. o.]), children and adolescents (3–15 y. o.), adults (16–64 y. o.), and elderly patients (>64 y. o.).

The most recent available trough serum IgG levels achieved on Privigen® were collected and, if applicable, compared with the IgG levels achieved on previous IgG treatment. SBIs were defined according to the diagnostic criteria of the US Food and Drug Administration (FDA) [13]; other infections were defined at the discretion of the investigators. Annualized rates per patient were calculated for SBIs, other infections, any infection, days spent in hospital, days missed from performing normal daily activities due to infection, and days on antibiotics for prophylaxis or treatment of infection. Days spent in hospital by retired or unemployed adult patients were considered as missed from performing normal daily activities. Data of one patient hospitalized during Privigen® treatment due to severe thrombocytopenia and of another patient hospitalized without a report of infection were included in the calculation of the relevant annualized rates to reflect the real-life characteristics of patients.

Tolerability of Privigen® was analyzed in all patients, including the infant patients with SCID. Privigen® infusion rate in each patient was calculated by dividing a single Privigen® dose by the entire duration of infusion. Percentages of patients who experienced at least one adverse event (AE) or serious AE (SAE), categorized by relatedness to Privigen®, were calculated. AE and SAE rates per infusion were not calculated due to missing data (exact number of events for each patient).

### Statistical Methods

All data were analyzed by descriptive statistics, including the mean, standard deviation (SD), median and range. Patients with missing data were excluded from the corresponding analyses, but included in other analyses, for which the data were available.

Annualized rates were calculated per patient per year (365 days), based on the total exposure to Privigen® of the patients included in each analysis. The annualized rate of SBIs was calculated along with its upper one-sided 99 % confidence interval (CI). Two-sided 95 % CIs were calculated for annualized rates of other infections and any infection. All CIs were calculated by the method described by Ulm et al. [14]. Correlation between the change in monthly IgG dose and the change in IgG levels was analyzed by linear regression.

## Results

### Patients

A total of 72 patient records were collected at 6 sites in Europe and the US. Three records of infants with SCID were analyzed separately (except for tolerability; see also Discussion). These included two boys and one girl with the median age of 3 months at the start of Privigen® treatment.

The remaining 69 patients included both male and female patients (Table I). The age at start of Privigen® treatment ranged from 0.1 years (1.3 months) to 90.0 years, with a median age of 38.0 years. The study included 23 (33.3 %) pediatric patients who were under 16 y. o. at the start of Privigen® treatment. Patients with either PID (*n* = 57; 82.6 %) or SID secondary to cancer were included. The most common diagnoses at the start of Privigen® treatment were IgG subclass deficiency (*n* = 18; 26.1 %; diagnosis by the referring physician), XLA (*n* = 13; 18.8 %), CVID (*n* = 10; 14.5 %), and chronic lymphocytic leukemia (CLL; *n* = 8; 11.6 %). Fourteen patients with IgG subclass deficiency also had specific antibody deficiency, i.e., poor response to *Streptococcus pneumoniae* vaccine and recurrent infections despite optimal therapy. Patients with XLA were present in all age groups except for the elderly. Patients with CVID or IgG subclass deficiency were observed mainly in the adult and elderly age classes. SID was observed in adult and elderly patients only.

**Table I:** Patient demographics and diagnosis at the start of Privigen®

	All patients		Infants (0–2 y. o.)		Children and adolescents (3–15 y. o.)		Adults (16–64 y. o.)		Elderly (>64 y. o.)	
Number of patients, n (%)	69	(100.0)	6	(8.7)	17	(24.6)	32	(46.4)	14	(20.3)
Male gender, n (% of the age group)	36	(52.2)	5	(83.3)	11	(64.7)	13	(40.6)	7	(50.0)
Age [years] at start of Privigen®, median (range)	38.0	(0.1–90.0)	1.5	(0.1–2.0)	9.0	(3.0–15.0)	52.0	(16.0–64.0)	71.5	(65.0–90.0)
Body weight [kg] at start of Privigen®, median (range)	65.9	(5.6–122.8)	8.9	(5.6–11.5)	28.0	(11.5–90.1)	73.5	(40.0–122.0)	83.2	(36.0–122.8)
Diagnosis, n (%)										
Any PID	57	(82.6)	6	(8.7)	17	(24.6)	26	(37.7)	8	(11.6)
IgG subclass deficiency	18	(26.1)	0	–	1	(1.4)	10	(14.5)	7	(10.1)
XLA	13	(18.8)	3	(4.3)	5	(7.3)	5	(7.3)	0	–
CVID	10	(14.5)	0	–	4	(5.8)	6	(8.7)	0	–
Hypogammaglobulinemia	6	(8.7)	0	–	1	(1.4)	4	(5.8)	1	(1.4)
SCID	3	(4.3)	n/a^a^	–	3	(4.3)	0	–	0	–
Other^b^	7	(10.1)	3	(4.3)	3	(4.3)	1	(1.4)	0	–
Any SID	12	(17.4)	0	–	0	–	6	(8.7)	6	(8.7)
CLL	8	(11.6)	0	–	0	–	3	(4.3)	5	(7.3)
MM	2	(2.9)	0	–	0	–	1	(1.4)	1	(1.4)
FL	1	(1.4)	0	–	0	–	1	(1.4)	0	–
ALL	1	(1.4)	0	–	0	–	1	(1.4)	0	–

ALL acute lymphoblastic leukemia, CLL chronic lymphocytic leukemia, CVID common variable immunodeficiency, FL follicular lymphoma, IgE immunoglobulin E, IgG immunoglobulin G, MM multiple myeloma, n number of patients, n/a not applicable, PID primary immunodeficiency, SCID severe combined immunodeficiency, SID secondary immunodeficiency, XLA X-linked agammaglobulinemia, y. o. years old

^a^ Infants with SCID (*n* = 3) were analyzed separately (Table IV)

^b^ Unspecified immunodeficiency, unspecified immune disorder, Wiskott-Aldrich syndrome, profound combined immunodeficiency, GATA-2 deficiency or hyper-IgE syndrome, each in <3 (<4.3 %) patients

Three non-infant patients with SCID underwent hematopoietic stem cell transplantation (HSCT) at least 6 months before the end of the Privigen® treatment. Two adult patients with SID received allogeneic bone marrow transplantation, one before and one during the Privigen® treatment.

### IgG treatment

All patients received individual Privigen® doses of 196–1,000 mg/kg per dosing cycle. All but two patients (*n* = 67; 97.1 %) were treated with Privigen® every 3 or 4 weeks. An adolescent patient with CVID and an adult patient with unspecified hypogammaglobulinemia received Privigen® biweekly at 555 mg/kg and 1,000 mg/kg, respectively. Both these patients were hospitalized for extended periods of time indicating a severe form of disease. The mean ± SD duration of Privigen® treatment for the entire cohort at the time of the chart review was 22.0 ± 15.0 months (median: 18.0 months, range: 3–60 months). This suggests that the majority of patients were at steady-state Privigen dosing at the time of collecting data for this report. The mean ± SD monthly Privigen® dose in all patients was 532 ± 250 mg/kg/month (Table II). Mean monthly doses were similar across all age groups, with the lowest mean dose in infants (481 mg/kg/month) and the highest mean dose in children and adolescents (606 mg/kg/month).

**Table II:** Dosing and administration of Privigen® and previous IgG treatment

	All patients		Infants (0–2 y. o.)		Children and adolescents (3–15 y. o.)		Adults (16–64 y. o.)		Elderly (>64 y. o.)	
Number of patients, n (%)	69	(100.0)	6	(8.7)	17	(24.6)	32	(46.4)	14	(20.3)
Privigen® monthly dose [mg/kg/month], mean (SD)	532	(250)	481	(137)	606	(230)	522	(307)	483	(133)
Range, min–max	196–2000		360–731		333–1110		196–2000		354–833	
Privigen® regimen, n (%)										
Q4W	59	(85.5)	4	(5.8)	15	(21.7)	26	(37.7)	14	(20.3)
Q3W	8	(11.6)	2	(2.9)	1	(1.4)	5	(7.2)	0	–
Q2W	2	(2.9)	0	–	1	(1.4)	1	(1.4)	0	–
Average infusion rate [mL/kg/h], mean (SD)	1.46	(0.58)	1.58	(0.54)	1.66	(0.38)	1.43	(0.67)	1.26	(0.49)
IgG-pretreated, n (%)	35	(50.7)	0	–	10	(14.5)	17	(24.6)	8	(11.6)
IVIG^a^	27	(39.1)	0	–	7	(10.1)	15	(21.7)	5	(7.2)
SCIG^a^	8	(11.6)	0	–	3	(4.3)	2	(2.9)	3	(4.3)

IgG immunoglobulin G, IVIG intravenous immunoglobulin, n number of patients, Q*x*W every *x* weeks, SCIG subcutaneous immunoglobulin, SD standard deviation, y. o. years old

^a^ Patients with >1 previous line of treatment were classified by the last IgG product received before Privigen®

Thirty-five (50.7 %) patients had been treated with other IgG products before switching to Privigen®. The change in the mean monthly IgG dose upon switching to Privigen® was not statistically significant (536 ± 320 mg/kg/month on Privigen® vs. 483 ± 204 mg/kg/month on previous IgG products; *p* = 0.4; *n* = 34). One patient with severe disease had an increase in IgG dose of 1,154 mg/kg/month, which strongly influenced the mean. Reported reasons of switching from other IVIG products to Privigen® included change in insurance coverage or product availability (*n* = 6), systemic reactions (*n* = 2), smaller volume and shorter administration times (*n* = 1), and frequent infection with the previous IVIG products (*n* = 1). Eight (11.6 %) patients switched to Privigen® from SCIG due to local reactions (*n* = 3), change in insurance coverage (*n* = 2), systemic reactions (*n* = 1) or difficulties with home-based infusions in pediatric patients (*n* = 1). Monthly IgG doses were the same on Privigen® as on previous SCIG (545 ± 254 mg/kg/month vs. 542 ± 244 mg/kg/month on previous SCIG). At the end of the observation period, 8 (11.6 %) patients switched from Privigen® to SCIG and 1 patient switched to a combination of Privigen® and SCIG.

The mean ± SD duration of the entire Privigen® infusion, including initial slow rates and a ramp-up, was 212 ± 59 min (median: 210 min, range: 90–392 min; *n* = 66), resulting in the median average infusion rate of 1.32 mL/kg/h (range: 0.73–3.64 mL/kg/h). The median infusion rates in pediatric patients were slightly higher than those in adults and elderly patients (Table II).

The majority of patients (*n* = 60; 87.0 %), including all patients with SID, received Privigen® at a hospital, the doctor’s office, or an infusion center. Six (8.7 %) patients received their treatment at home, and 3 (4.3 %) patients received their treatment both at home and at a hospital or infusion center.

### Effectiveness

**Fig. 1:**
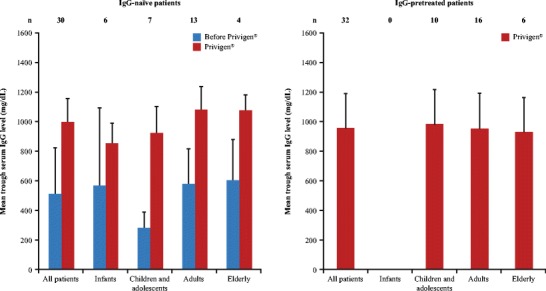
Trough serum IgG levels achieved with Privigen® by age class and IgG-pretreatment. IgG-naïve (*n* = 30) and IgG-pretreated (*n* = 32) patients with available serum IgG levels both before and after the start of Privigen® were analyzed by age class: infants (0–2 y. o.), children and adolescents (3–15 y. o.), adults (16–64 y. o.) and elderly patients (>64 y. o.). The mean values of the most recent available trough serum IgG levels are shown along with the SD (error bars) and the number of patients in each subgroup (n). Please note that there were no IgG-pretreated infants in this study

**Fig. 2:**
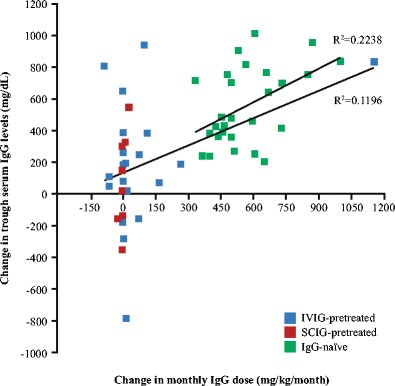
Changes in monthly IgG dose and trough serum IgG levels. Patients with available serum IgG levels both before and after the switch to Privigen® were analyzed, including IgG-naïve (*n* = 27; green), IVIG-pretreated (blue; *n* = 23) and SCIG-pretreated (red; *n* = 8) patients. Three IgG-naïve infants were excluded from this analysis due to high content of maternal IgG before the start of the Privigen® treatment. The change in trough serum IgG levels was plotted against the absolute monthly Privigen® dose (IgG-naïve patients) or against the change in monthly IgG dose (IgG-pretreated patients). An IVIG-pretreated patient with the highest increase of monthly IgG dose received an unusually high monthly Privigen® dose (2,000 mg/kg/month) due to a severe form of disease. Correlation was analyzed by calculating the linear regression coefficients (R) for IgG-naïve and IgG-pretreated patients (IVIG- and SCIG-pretreated combined)

Effectiveness was analyzed separately in the main cohort of patients (*n* = 69) and in infants with SCID (*n* = 3). In the main cohort, the mean ± SD of the most recent available trough serum IgG levels achieved on Privigen® was 970 ± 248 mg/dL (*n* = 63; median: 954 mg/dL; range: 407–1,581 mg/dL; Table III). Only two (2.9 %) patients had confirmed trough serum IgG levels of less than 500 mg/dL, both with SID secondary to cancer. The mean trough IgG levels on therapy were similar across all age classes, as well as between the IgG-pretreated and IgG-naïve patients (Fig. 1). As expected, IgG levels increased in previously untreated patients (*n* = 27, three patients with substantial levels of maternal IgG at the start of Privigen® treatment were excluded from this analysis; data were not available in three other IgG-naïve patients). The median increase in IgG levels in these patients was 447 mg/dL (range: 201 mg/dL to 1,014 mg/dL). However, the change in individual patients was poorly correlated with the monthly IgG dose (Fig. 2). Similarly, no consistent change in serum IgG concentrations was seen in IVIG-pretreated patients (*n* = 23; median change: 165 mg/dL, range: −784 mg/dL to 936 mg/dL). SCIG-pretreated patients showed a wide variety of IgG level changes despite almost unchanged monthly IgG doses (*n* = 8; median change: 84 mg/dL, range: −350 mg/dL to 542 mg/dL).

**Table III:** Effectiveness of Privigen® treatment

	All patients	Infants (0–2 y. o.)	Children and adolescents (3–15 y. o.)	Adults (16–64 y. o.)	Elderly (>64 y. o.)	Patients with severe disease	Patients without severe disease
Number of patients, n (%)	69 (100.0)	6 (8.7)	17 (24.6)	32 (46.4)	14 (20.3)	2 (2.9)	67 (97.1)
Most recent available trough serum IgG levels, mean (SD)^a^	970 (248)	856 (188)	960 (211)	1011 (244)	938 (335)	1238 (371)	961 (243)
SBIs, n (number of events)	10 (11)	0	3 (3)	5 (6)	2 (2)	1 (2)	9 (9)
Annualized rate [events/patient/year]	0.087	0	0.112	0.093	0.086	0.392	0.074
Upper one-sided 99 % CI	0.170	0	0.375	0.225	0.363	1.649	0.155
Other infections, n (number of events)	44 (126)	5 (14)	11 (33)	22 (62)	6 (17)	2 (6)	42 (120)
Annualized rate [events/patient/year]	0.995	1.180	1.230	0.957	0.734	1.177	0.988
Two-sided 95 % CI	0.829–1.185	0.645–1.981	0.847–1.729	0.725–1.223	0.428–1.176	0.432–2.562	0.819–1.818
Any infection, n (number of events)	46 (137)	5 (14)	12 (36)	23 (68)	6 (19)	2 (8)	44 (129)
Annualized rate [events/patient/year]	1.082	1.180	1.342	1.050	0.821	1.569	1.062
Two-sided 95 % CI	0.909–1.279	0.645–1.981	0.941–1.859	0.815–1.331	0.494–1.282	0.678–3.092	0.866–1.262
Days in hospital due to infection, n (total days)	18 (460)^b^	2 (47)	10 (278)^b^	5 (130)^b^	1 (5)	2 (303)	16 (157)
Annualized rate [events/patient/year]	3.63^b^	3.96	10.36^b^	2.01^b^	0.22	59.44	1.29
Days missed from performing normal daily activities due to infection, n (total days)	23 (563)^b^	2 (63)	10 (316)^b^	10 (179)^b^	1 (5)	2 (303)	21 (260)
Annualized rate [events/patient/year]	4.45^b^	5.31	11.78^b^	2.76^b^	0.22	59.44	2.14
Days on antibiotics due to prophylaxis or infection, n (total days)^c^	40 (5223)	6 (545)	13 (2577)	16 (1662)	5 (439)	1 (50)	39 (5173)
Annualized rate [events/patient/year]	47.0	45.9	96.1	32.6	20.5	17.1	47.8

CI confidence interval, IgG immunoglobulin G, n number of patients, SBI serious bacterial infection, SD standard deviation, w/o without, y. o. years old

^a^ Data not available in 6 patients (*n* = 63, including 6 infants, 17 children and adolescents, 29 adults and 11 elderly patients)

^b^ Total numbers of events and the annualized rates were strongly influenced by 2 patients with severe disease (a child and an adult)

^c^ Data not available in 7 patients (*n* = 62, including 6 infants, 17 children and adolescents, 27 adults and 12 elderly patients)

Ten (14.5 %) patients had a total of 11 SBIs, resulting in an annualized SBI rate of 0.087 events/patient/year (upper one-sided 99 % CI: 0.170; Table III). The most common SBI was pneumonia (*n* = 8; 11.6 %; 0.063 events/patient/year). Other SBIs observed were bacteremia/sepsis (*n* = 2; 2.9 %) and visceral abscess (*n* = 1; 1.4 %). Most common other infections were (rhino-) sinusitis (*n* = 24; 34.8 %) and upper respiratory tract infection (URI; *n* = 10; 14.5 %). One patient had documented recurrent URI that was considered one infection. The annualized SBI rates in pediatric (*n* = 23) and adult/elderly (*n* = 46) patients were 0.078 events/patient/year and 0.091 events/patient/year, respectively. The annualized rate of any infection in all patients was 1.082 events/patient/year (two-sided 95 % CI: 0.909–1.279); in pediatric and adult/elderly patients the rates were 1.215 events/patient/year and 0.899 events/patient/year, respectively.

Patients spent an average of 3.63 days/patient/year in hospital due to infection. However, 303 of 460 days were attributed to two patients with severe disease. An adolescent patient with CVID was hospitalized for a total of 210 days due to “atypical pneumonia”, sinusitis and gastroenteritis caused by *Salmonella* spp. An adult patient with hypogammaglobulinemia was hospitalized for a total of 93 days due to two episodes of SBI (intestinal abscess and bacteremia caused by *Campylobacter* spp.). The annualized rate of hospitalization in the remaining children and adolescents was 2.85 days/patient/year and in adults 0.59 days/patient/year. Patients with SCID included into the main cohort were hospitalized on average for 0.89 days/patient/year. Over two-thirds of the patient cohort (*n* = 51; 73.9 %) were not hospitalized due to infection during Privigen® treatment.

The annualized rate of days missed from performing normal daily activities due to infection was 4.45 days/patient/year. This rate was considerably lower upon exclusion of the patients with severe disease (remaining patients: *n* = 67; 2.14 days/patient/year). Forty (58.0 %) patients were treated with antibiotics for prophylaxis or treatment of infection, including 12 (16.7 %) patients who were continuously receiving antibiotics while on Privigen®. The annualized rate of antibiotic treatment based on total exposure of all patients to Privigen® was 47.0 days/patient/year. Antibiotic use was much more frequent in children and adolescents than in adults and elderly patients (Table III).

#### IgG Treatment and Effectiveness in Infant Patients With SCID

Treatment schedules and effectiveness of Privigen® in infants with SCID (*n* = 3) were analyzed separately. These patients were started on Privigen® at 2–4 months of age and either did not undergo HSCT (*n* = 1) or underwent the transplantation shortly before starting Privigen® (*n* = 2). One patient with two episodes of gram-negative bacteria sepsis was treated with 1,000 mg/kg Privigen® once weekly, resulting in a monthly IgG dose of 4,000 mg/kg/month. A second patient had disseminated *Pneumocystis jirovecii* infection and was treated with 2,000 mg/kg Privigen® every 3 weeks (2,700 mg/kg/month). The third patient was treated with 400 mg/kg Privigen® every 3 weeks (532 mg/kg/month). In spite of the high monthly doses of Privigen®, trough serum IgG levels in these patients were similar to the main patient cohort (Table IV). The patients experienced a total of 6 SBIs, including bacteremia/sepsis (*n* = 2), septic arthritis, and pneumonia (each *n* = 1). Two of these patients were hospitalized for the whole period of Privigen® treatment, resulting in an overall hospitalization rate of 279.3 days/patient/year. The patients were treated with antibiotics for the whole period of hospitalization.

**Table IV:** Privigen® dosing and effectiveness in infants with SCID

	Mean, range or n	Annualized rate (events/patient/year)	CI
Privigen® monthly dose [mg/kg/month], mean (SD)	2411 (1752)	n/a	n/a
Range, min–max	532–4000	n/a	n/a
Most recent available trough serum IgG levels, mean (SD)^a^	1018 (182)	n/a	n/a
SBIs, n (number of events)	3 (6)	5.319	12.917^b^
Other infections, n (number of events)	1 (2)	1.773	0.215–6.404^c^
Any infection, n (number of events)	3 (8)	7.092	3.062–13.973^c^
Days in hospital due to infection, n (total days)	3 (315)	279.2	n/a
Days missed from day care due to infection, n (total days)	3 (315)	279.2	n/a
Days on antibiotics due to prophylaxis or infection, n (total days)	3 (315)	279.2	n/a

CI confidence interval, IgG immunoglobulin G, n number of patients, n/a not applicable, SBI serious bacterial infection, SD standard deviation

^a^ Data not available in 1 patient (*n* = 2)

^b^ Upper one-sided 99 % CI

^c^ Two-sided 95 % CI

### Tolerability

The occurrence of AEs was recorded in all patients, including infants with SCID (*n* = 72). Thirteen (18.1 %) patients experienced at least one AE, including 10 (13.9 %) patients with AEs possibly related to Privigen®. The most common related AEs were headache after infusion (*n* = 6; 8.3 %), fever, and chills or rigors (each *n* = 2; 2.8 %; Table V). One SAE of swelling of the legs after Privigen® infusion was reported in an adult patient with IgG subclass deficiency and was considered not related to Privigen®. No other SAEs were reported (excluding infections reported under effectiveness). One patient with SCID died at the age of 6.5 months due to severe viral infection, before receiving HSCT.

**Table V:** Tolerability of Privigen® treatment (all patients including infants with SCID)

Preferred term	All events		At least possibly related to Privigen®	
	Number of patients	Percent	Number of patients	Percent
Any AE	13	18.1	10	13.9
Headache after infusion	6	8.3	6	8.3
Fever	2	2.8	2	2.8
Chills or rigors	2	2.8	2	2.8
Fatigue	1	1.4	1	1.4
Syncope during or after infusion	1	1.4	1	1.4
Emesis	1	1.4	1	1.4
Increased serum creatinine	1	1.4	1	1.4
Diarrhea	1	1.4	0	0
Neuropathy	1	1.4	0	0
Any SAE	1	1.4	0	0
Swelling of lower limbs after infusion	1	1.4	0	0

AE adverse event, SAE serious adverse event

The majority of patients (*n* = 61; 84.7 %) did not have any non-infectious complications of the underlying disease during Privigen® treatment. One patient experienced a combination of transfusion-dependent, steroid-resistant Diamond-Blackfan anemia, iron overload and chronic enteroviral infection. No cases of Privigen®-related anemia (positive Coombs test), changes in liver or renal function, or accumulation of proline were reported.

## Discussion

This study shows that Privigen® doses and regimens previously tested in prospective clinical studies were also used in clinical practice, which allows a valid comparison of the real-life effectiveness and tolerability with the clinical studies results.

The minimal duration of the Privigen® treatment was set for 3 months, which ensured that the majority of patients were at steady-state Privigen® dosing at the time of collecting data for this report. The mean trough IgG levels achieved in this study and resulting from doses recommended in the respective Privigen® labels closely matched previously published results of clinical studies (811–1,027 mg/dL [8, 9]) and other IVIG products (830–1,120 mg/dL [11, 15–17]). The vast majority of the patients reached IgG levels above 500 mg/dL, with a mean trough serum IgG level of 970 mg/dL for the main cohort. Indeed, higher IgG levels have been shown to be associated with lower frequencies of infections [18, 19], and the incidence of pneumonia we observed fell within the 95 % CI for the corresponding IgG level (0.008–0.068 events/patient/year) in the pooled analysis by Orange et al. [18]. The median IgG levels were consistent with contemporary guidelines that aim to achieve at least 700–800 mg/dL. Observed variability of individual IgG levels of more than 3.5 fold (407–1,581 mg/dL) supports the recent findings that ‘protective’ IgG levels are highly individual [19–21]. The annualized SBI rate observed in this study fell into the range reported in clinical studies (0.080–0.120 events/patient/year [8, 9]). The annualized rate of any infection in clinical practice was lower than in clinical studies (3.55–3.79 events/patient/year [8, 9]), which may be partially attributed to the retrospective nature of this study, where some episodes of mild infections might not have been recorded, unlike clinical trials in which symptoms are entered into diaries every day. Another possible factor was the inclusion of patients with IgG subclass deficiency, with or without antibody production, that are commonly excluded from clinical trials. The annualized rate of hospitalization was above the range determined in clinical studies (0.33–2.31 days/patient/year [8, 9]). This result was caused by the presence of two patients with severe disease in the cohort. Such patients have been previously reported and may have been excluded from clinical studies or analyzed separately [12]. The rest of the patients demonstrated hospitalization rates within the clinical study range. Despite the higher hospitalization rate, the annualized rate of days missed from performing normal daily activities was lower than in the clinical studies (7.94–8.97 days/patient/year [8, 9]), but these data may have been underestimated due to the retrospective nature of the study.

We observed high variability in the IgG levels achieved with Privigen® treatment in different IgG-naïve patients, which was poorly correlated with the monthly IgG dose but consistent with the observations of Lucas et al. [20] on the variability of doses required to achieve desired IgG levels in different patients. Switching from a previous IgG product to Privigen® resulted in a similar variety of IgG level changes. These findings are in line with a recent analysis that demonstrated high variability of the relationship between IgG levels and IgG dose in individual patients with PID [22]. In addition, the present study included patients whose immunoglobulin levels could be expected to be variable over time (such as patients with multiple myeloma), and interventions that may have influenced serum IgG levels, such as bone marrow transplantation or HSCT, were not prohibited. This study also included patients who switched from SCIG to IVIG (Privigen®), from Privigen® to SCIG and from other IVIG products to Privigen®. Thus, our observations highlight the importance of individualized choice of treatment and the necessity of targeting a certain clinical response (e.g., level of protection against infections), rather than predefined serum IgG levels, when selecting the dosing regimen. The target of treatment may also change over time, depending on the patient’s preference and clinical condition.

Age class analysis showed high similarity of Privigen® doses and effectiveness between all age groups, with the exception of more frequent hospitalization and antibiotic use in pediatric patients than in adult and elderly patients. These results are consistent with the similarity of results obtained in clinical studies with pediatric and adult patients [5, 8, 9].

Unlike registration clinical studies, this study also included patients with SID. These patients were treated with identical doses of Privigen® administered at a hospital or infusion center. Remarkably, inclusion of patients with SID did not prevent the results of this study from matching those of clinical studies, suggesting similar effectiveness and tolerability of Privigen® in SID and PID in clinical practice. One limitation of this result is that all patients with SID were treated at the same clinical site. Nevertheless, the tolerability observed in our study was very similar to the results of another single-center study in patients with SID [23].

We analyzed the dosage and effectiveness of Privigen® in infants with SCID separately from the main patient cohort due to potential differences in their disease mechanisms. Patients with SCID before and shortly after HSCT may lack cell-mediated immunity, a defect that cannot be rescued by replacement IgG therapy. This is one of the reasons why such patients are usually not included in clinical studies of IgG products. Indeed, these patients demonstrated a distinct treatment and outcome pattern: two of these patients received exceptionally high monthly IgG doses of 2,700–4,000 mg/kg/month, and all three were hospitalized and treated with antibiotics for extended periods of time.

Privigen® was well tolerated in clinical practice. The absence of Privigen®-related SAEs and the percentage of infusion-related AEs, with headache being the most common related AE, matched the results of recent clinical studies with Privigen® (18 % [8, 9]) and other IVIG products (14 % to 20 % [11, 15–17]).

## Conclusions

This study demonstrates that the effectiveness and tolerability of Privigen® previously observed in clinical studies are also evident in clinical practice in Europe and the US, in both adult and pediatric patients. Few exceptions in Privigen® dosage and effectiveness were observed in infant patients with SCID and patients with severe disease.
